# Supplementary material for: Comparative Transcriptome Analysis Reveals Novel Candidate Resistance Genes Involved in Defence against Phytophthora cactorum in Strawberry
Source: Int J Mol Sci. 2023 Jun 29;24(13):10851. doi: 10.3390/ijms241310851 (PMC10341869; doi:10.3390/ijms241310851)
Supplement: Supplementary file 1 [file ijms-24-10851-s001.zip › Supplementary Figures.pdf]

Comparative Transcriptome Analysis Reveals Novel Candidate Resistance Genes Involved in Defence Against *Phytophthora cactorum* in Strawberry

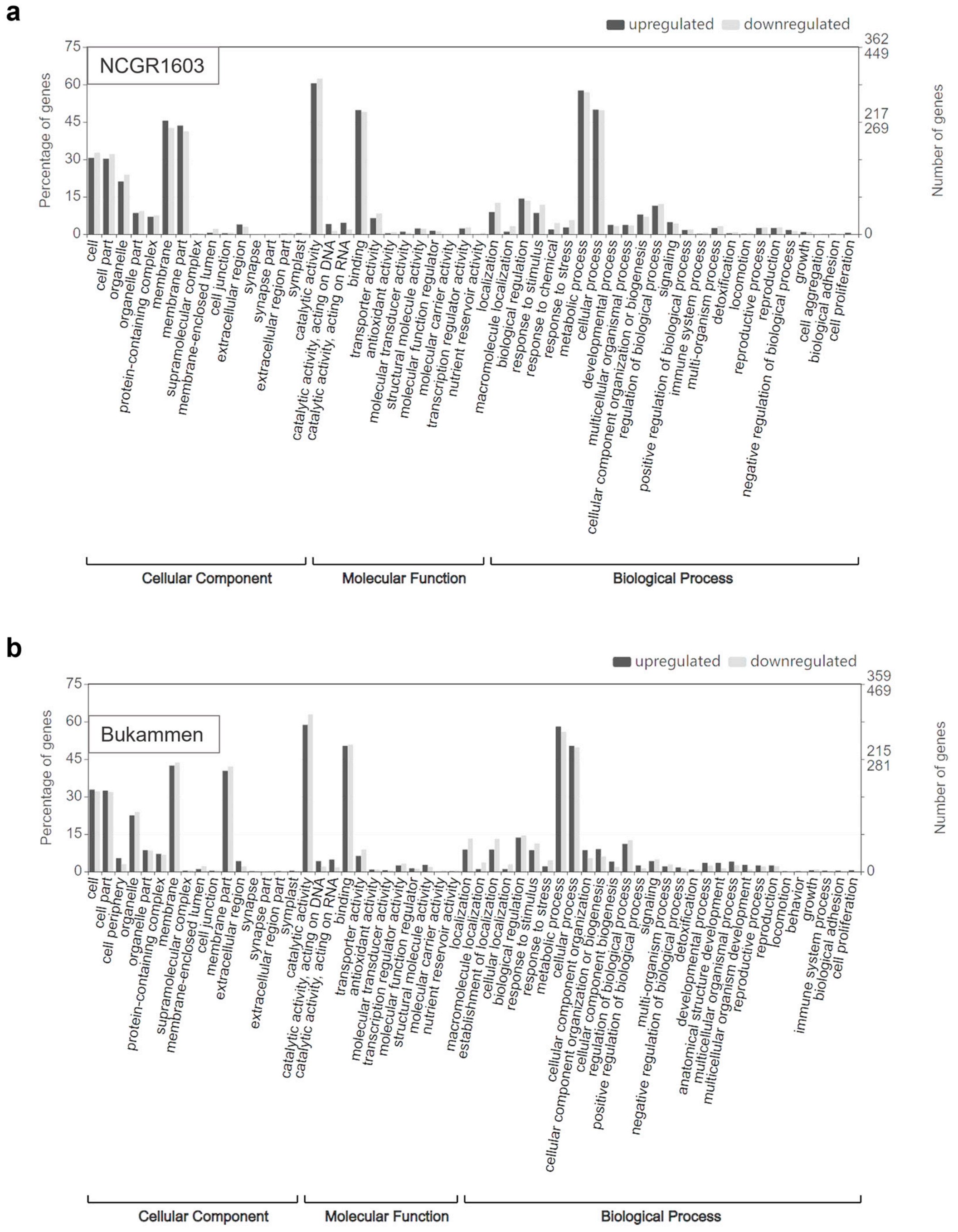

Supplementary Figure S1. Gene Ontology (GO) analysis of the differentially expressed genes (DEGs) in the resistant *Fragaria vesca* genotypes (a) NCGR1603 and (b) Bukammen, relative to the inoculated susceptible genotype NCGR1218, 48 h after inoculation with *Phytophthora cactorum*. The left y-axis indicates the percentage of genes representing each category. The right y-axis indicates the total number of genes in a category.

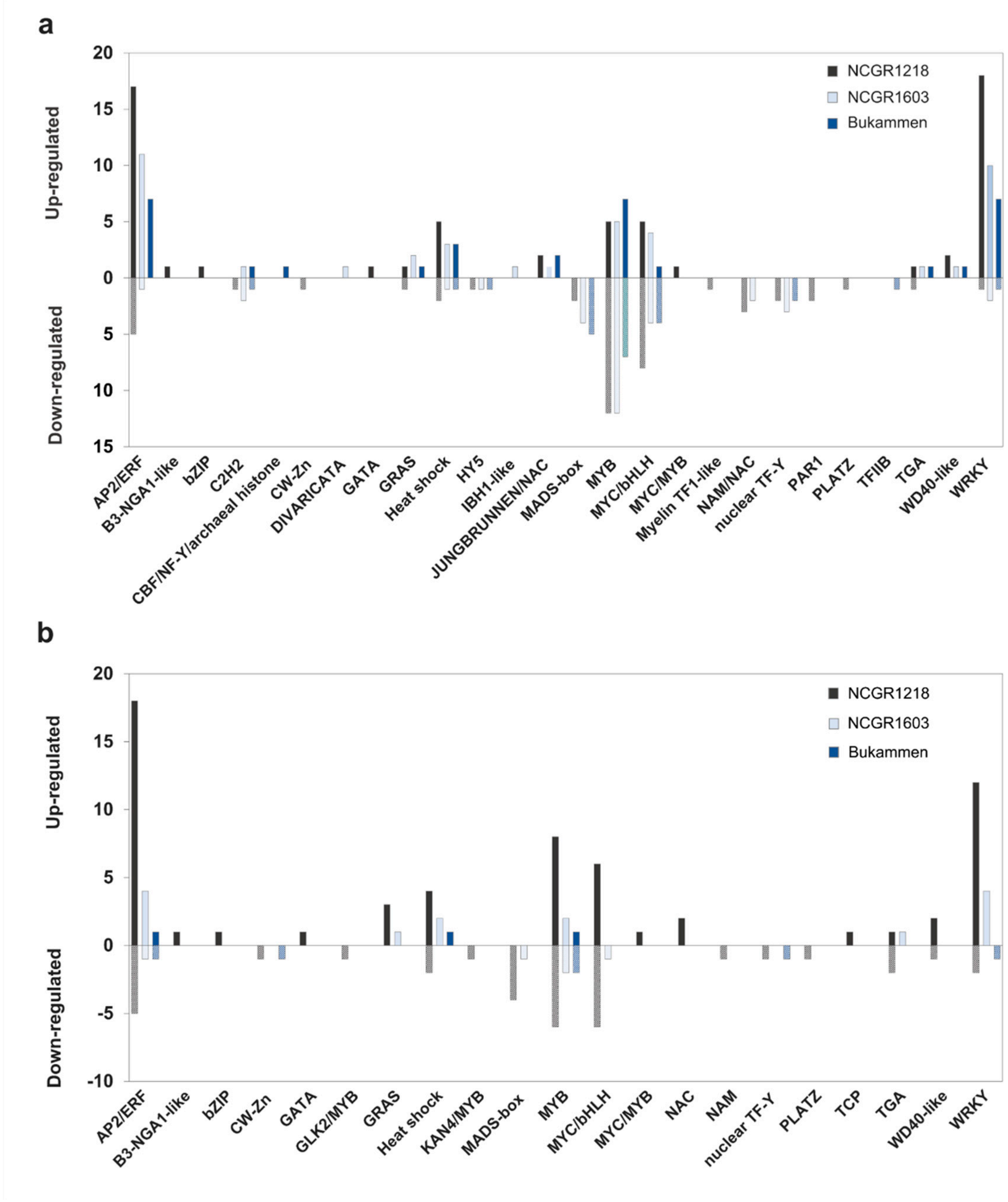

Supplementary Figure S2. Number of differentially expressed transcription factor (TF) genes in *Fragaria vesca* genotypes in response to *Phytophthora cactorum* inoculation. **a.** The number of upregulated and downregulated transcription factor genes in different TF families in the susceptible genotype NCGR1218 and the two resistant genotypes NCGR1603 and Bukammen, 48 h after inoculation with *P. cactorum,* relative to their untreated control. **b.** The number of upregulated and downregulated transcription factor genes in the susceptible and resistant genotypes 48 h after inoculation with *P. cactorum*, relative to their wounded (mock) control. Genes were considered differentially expressed when fold change was ≥ 2 or ≤ -2 with $p < 0.05$.

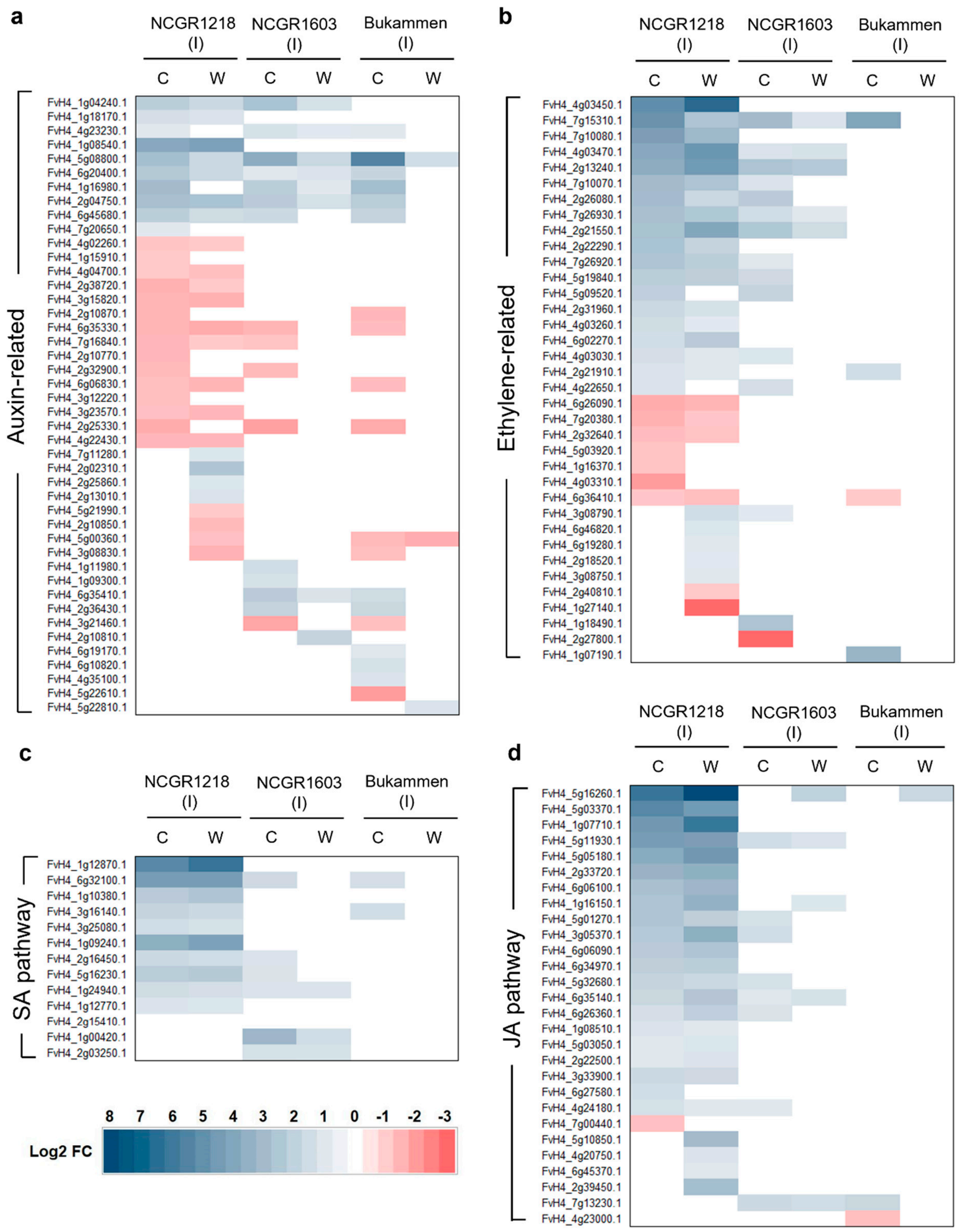

Supplementary Figure S3. Differentially expressed genes related to phytohormone signalling pathways in *Fragaria vesca* genotypes in response to inoculation with *Phytophthora cactorum* after wounding. **a.** Auxin related genes (biosynthesis, transport, and signalling). **b.** Salicylic acid pathway. **c.** Biosynthesis and signalling of ethylene. **d.** Jasmonic acid pathway. Blue and red indicates upregulated and downregulated genes, respectively (Log2 fold change (FC) ≥ 1 or ≤ -1; $p < 0.05$). White indicates no significant change in gene expression between inoculated (I) genotypes relative to their controls (C=untreated and W=wounded).

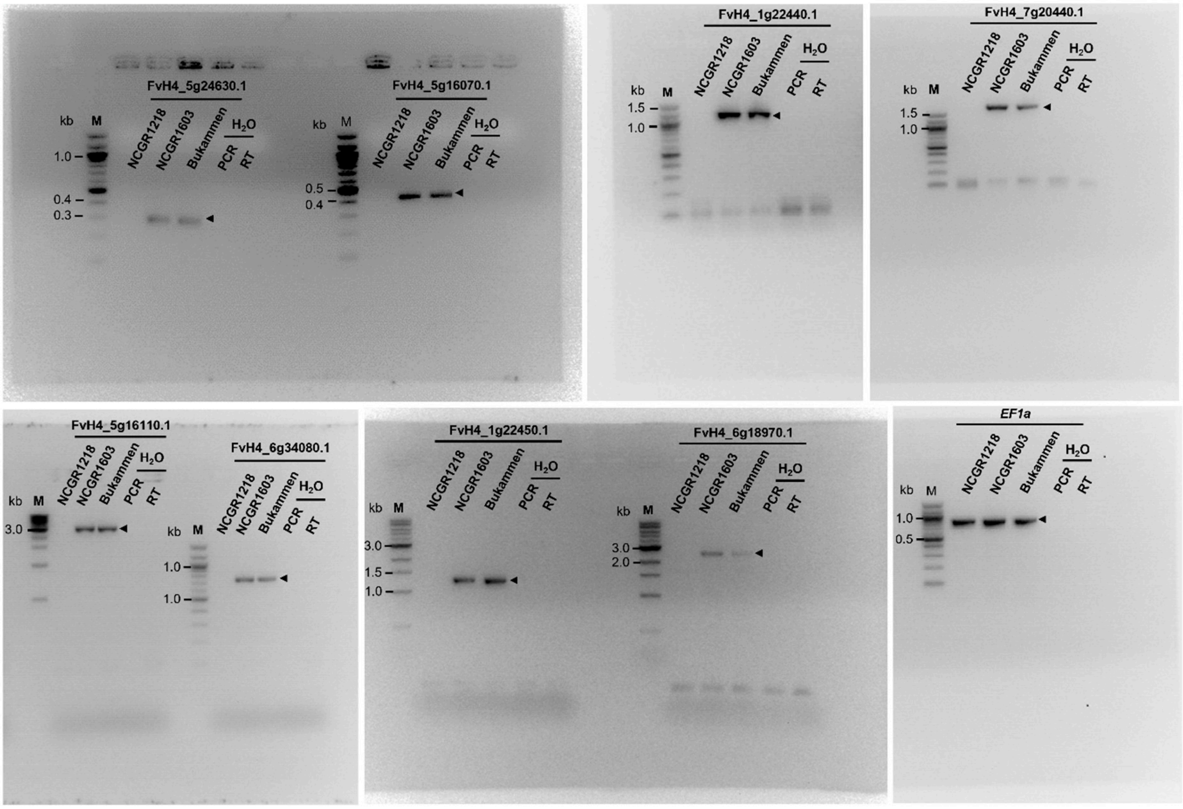

Supplementary Figure S4. Original agarose gel electrophoresis images of the RT-PCR amplified *Fragaria vesca* transcripts of the resistant genotypes NCGR1603 and Bukammen and the susceptible genotype NCGR1218 after inoculation with *Phytophthora cactorum*. Cropped gel images are displayed in Fig. 6. Black arrow indicates expected amplified transcript. M indicates molecular marker.

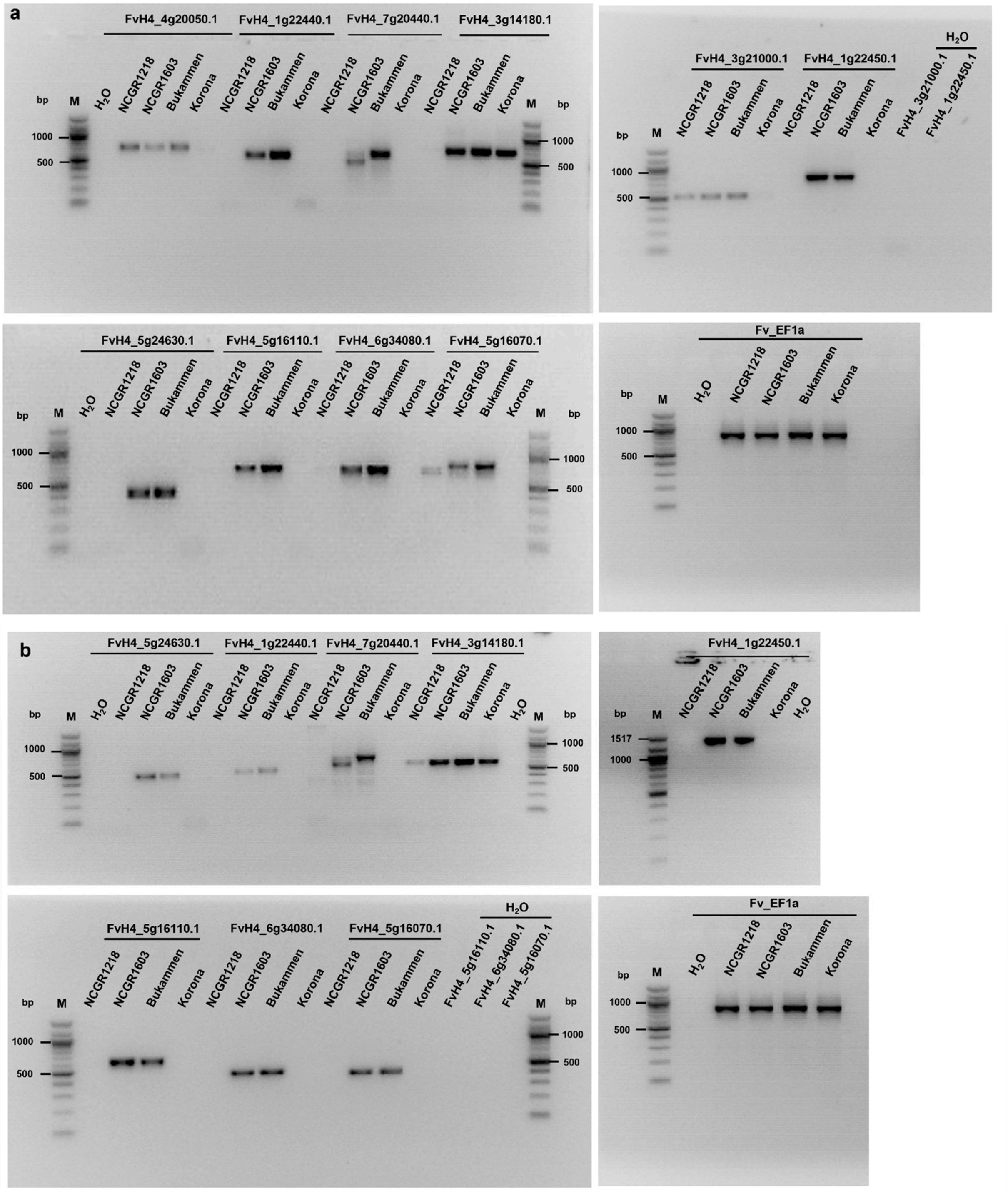

Supplementary Figure S5. Original agarose gel electrophoresis images of the PCR amplified fragments of *Fragaria vesca* genes of the resistant genotypes NCGR1603 and Bukammen, the susceptible genotype NCGR1218, and the moderately susceptible cultivar Korona (*F. x ananassa*). Cropped gel images are displayed in Fig. 7. M indicates molecular marker (100 bp).
