# Supplementary material for: Comparative Transcriptome Analysis Reveals Novel Candidate Resistance Genes Involved in Defence against Phytophthora cactorum in Strawberry
Source: Int J Mol Sci. 2023 Jun 29;24(13):10851. doi: 10.3390/ijms241310851 (PMC10341869; doi:10.3390/ijms241310851)
Supplement: Supplementary file 1 [file ijms-24-10851-s001.zip › Supplementary Table S1.pdf]

Supplementary Table S1. List of primers used for the present study

Primer Name[a]	Sequence (5′ – 3′)	Amplicon size (bp)	Annealing temperature (°C)
**For PCR**			
FvH4_5g24630-F1<br>FvH4_5g24630-R1	GTGGCGGTGTTTAGGTGTCT<br>TAGAACATTTTGCGCTCACG	411	60
FvH4_5g24630-F2<br>FvH4_5g24630-R2	GGTGTCTTCTTCGTGGTGGT<br>GATCAACTGCCTTGCGTACA	461	60
FvH4_4g20050-F<br>FvH4_4g20050-R	TCTCTTTCGTCGCTTCCTTC<br>CCAAACTGGAAAATCCCAAA	800	60
FvH4_7g05680-F<br>FvH4_7g05680-R	GAGGCAACACAACTGGTTCA<br>GAACTAATCGCGGAATCCAA	429	55
FvH4_1g22440-F1<br>FvH4_1g22440-R1	TCGGGAGTTCTCTTGCTGTT<br>TGAAGGTTGCAGAAGACACG	582	60
FvH4_1g22440-F2<br>FvH4_1g22440-R2	TTTGGATCTTCCCGATTACG<br>CCAACATTCCCCAAAAATTG	482	60
FvH4_6g53350-F<br>FvH4_6g53350-R	TTTCTATGGCTTCCCTGTCG<br>AGAACGGAAGTGCCATCATC	569	55
FvH4_7g20440-F<br>FvH4_7g20440-R	CTATGGCGAAAACACGGAAC<br>AGAGATGCCGGATCTGTGAA	593	60
FvH4_7g20440-F2<br>FvH4_7g20440-R2	CTGGTACGCGGAGGGTAGTA<br>CTCCTCGACGGTAGACTTGG	662	60
FvH4_3g14180-F1<br>FvH4_3g14180-R1	CAAGATGCTGCTCATTTGGA<br>CAGGGCATGACCTACACCTT	648	60
FvH4_3g14180-F2<br>FvH4_3g14180-R2	GGCAGCATAGTTGCAGTGAA<br>GCTCTGCCAAAGACACAACA	545	60
FvH4_5g16110-F1<br>FvH4_5g16110-R1	CAGAATGTGGTGGGTGAGTG<br>TTGGAAGGGACGATTACGAG	643	60
FvH4_5g16110-F2<br>FvH4_5g16110-R2	AACCTGCATTGTGGGATAGC<br>AAACCAAGCGTTGGTGAAAC	597	60
FvH4_6g34080-F1<br>FvH4_6g34080-R1	CGGAAAAGGGCAATCACTCT<br>ATCTTCTTGTGTCGGCATGG	624	60
FvH4_6g34080-F2<br>FvH4_6g34080-R2	TGGCAAGCGATCCACTTAAT<br>GGCAGGCCAATACAGTAACG	441	60
FvH4_5g16070-F1<br>FvH4_5g16070-R1	GCAGGAGGTTCAGAAAGGTG<br>GTTTTCAGCAAGGAGCCAAG	697	60
FvH4_5g16070-F2<br>FvH4_5g16070-R2	AAGCTGCGCCATATTGAACT<br>CAGCAACAAGGTCAGCAAAA	415	60
FvH4_3g21000-F<br>FvH4_3g21000-R	TTTCTAGCGTGACCGGTTCT<br>CTTCTTCCGAGCATTTTTGG	501	60
FvH4_1g22450-F<br>FvH4_1g22450-R	CCGGTACTCTGGTGAAGCAT<br>GGGGTGAAAAAGAGCAAACA	632	60
GW_FvH4_1g22450-F<br>GW_FvH4_1g22450-R	*GGGGACAAGTTTGTACAAAAAAGCAGGCT*ATGGATCCGTGGGTTGATGT<br>*GGGGACCACTTTGTACAAGAAAGCTGGGT*TTAAACATCCTCTGTCATAA	1363	60

Primer Name[a]	Sequence (5′ – 3′)	Amplicon size (bp)	Annealing temperature (° C)
EF1a-F[b]	CTGTAACAAGATGGATGCCACC	803	60
EF1a-R	GGCGCATGTCCCTCACAGCAA		
**For RT-PCR**			
GW_FvH4_1g22440-F	*GGGGACAAGTTTGTACAAAAAAGCAGGCT*ATGATGTCTTTAGCCAAAAC	1180	60
GW_FvH4_1g22440-R	*GGGGACCACTTTGTACAAGAAAGCTGGGT*CTATCTGCCGGTGGTGCCTT		
GW_FvH4_7g20440-F	*GGGGACAAGTTTGTACAAAAAAGCAGGCT*ATGGCGAAAACACGGAACA	1768	60
GW_FvH4_7g20440-R	*GGGGACCACTTTGTACAAGAAAGCTGGGT*GTCCTACTTAAGCCAGTCCT		
GW_FvH4_5g24630-F	*GGGGACAAGTTTGTACAAAAAAGCAGGCT*ATGGTGACTGTGTTTGGGCGT	292	60
GW_FvH4_5g24630-R	*GGGGACCACTTTGTACAAGAAAGCTGGGT*TCAGCCTCCATAGCCACGTAC		
GW_FvH4_6g34080-F	*GGGGACAAGTTTGTACAAAAAAGCAGGCT*ATGGAATATCACCAGTTCTA	721	60
GW_FvH4_6g34080-R	*GGGGACCACTTTGTACAAGAAAGCTGGGT*TTAATCTTCTTGTGTCGGCAT		
GW_FvH4_5g16110-F	*GGGGACAAGTTTGTACAAAAAAGCAGGCT*ATGCTGATTCTCGAAATACT	2905	60
GW_FvH4_5g16110-R	*GGGGACCACTTTGTACAAGAAAGCTGGGT*TCACGATTGAATTATTCCA		
GW_FvH4_5g16070-F	*GGGGACAAGTTTGTACAAAAAAGCAGGCT*TTGACCTTGCCTTCCTTCT	3228	60
GW_FvH4_5g16070-R	*GGGGACCACTTTGTACAAGAAAGCTGGGT*TCATGTGAACCGAAAAAGAT		
GW_FvH4_1g22450-F	*GGGGACAAGTTTGTACAAAAAAGCAGGCT*ATGGATCCGTGGGTTGATGT	1197	60
GW_FvH4_1g22450-R	*GGGGACCACTTTGTACAAGAAAGCTGGGT*TTAAACATCCTCTGTCATAA		
GW_FvH4_6g18970-F	*GGGGACAAGTTTGTACAAAAAAGCAGGCT*ATGGAGGCGCCGGGGTCCCT	2602	60
GW_FvH4_6g18970-R	*GGGGACCACTTTGTACAAGAAAGCTGGGT*TCATGTAGCCACCATGCGCG		

[a]Primer name refers to the *F. vesca* Hawaii v4.a1 gene ID; F and R indicate forward and reverse primers, respectively; GW indicates attB1 or attB2 sequences (in italics) from the Gateway cloning system. [b]*EF1a* gene (*Elongation factor 1 alpha*) primer sequences used in this study were obtained from Clancy *et al.*[1]
